# Comprehensive co-expression analysis reveals candidate regulatory genes associated with carcass and meat quality traits in Neijiang and Large White pigs

**DOI:** 10.5713/ab.25.0259

**Published:** 2025-06-24

**Authors:** Patrick Kofi Makafui Tecku, Dong Chen, Kai Wang, Shixin Yu, Jiamiao Chen, Guoqing Tang

**Affiliations:** 1 Key Laboratory of Livestock and Poultry Multi-omics, Ministry of Agriculture and Rural Affairs, College of Animal Science and Technology, Sichuan Agricultural University, Chengdu, China; 2 Farm Animal Germplasm Resources and Biotech Breeding Key Laboratory of Sichuan Province, College of Animal Science and Technology, Sichuan Agricultural University, Chengdu, China; 3 New Hope Liuhe Co., Ltd., Key Laboratory of Digital Intelligent Breeding Technological Innovation for Swine and Poultry, Ministry of Agriculture and Rural Affairs, Chengdu, China

**Keywords:** Carcass Trait, Key Gene, Meat Quality, Pig, Weighted Gene Co-expression Network Analysis (WGCNA)

## Abstract

**Objective:**

The Neijiang indigenous pig breed of China and the Western Large White pig breed have unique meat quality and carcass characteristics. However, the genetic factors and mechanisms influencing their distinct meat and carcass traits are still not well understood. Therefore, using weighted gene co-expression network analysis (WGCNA), this study aimed to identify key genes influencing these traits.

**Methods:**

Transcriptome data from 17 Neijiang and 22 Large White pigs, along with their carcass weight, backfat thickness, eye muscle area, meat color, and muscle pH phenotypic data, were analyzed using WGCNA. A total of 9,249 genes were used to construct a weighted gene co-expression network.

**Results:**

Twenty-two co-expression gene modules were identified. Genes in the top modules were enriched in processes relevant to carcass and meat quality, such as protein transport. Further analysis identified six key genes, including *HSPH1*, *HSPA4*, *DNAJA4*, *MRPL3*, *SEC63*, and *SRP54*, for the Neijiang breed. Also, five key genes, consisting of *EP300*, *SETD2*, *NIPBL*, *NAT10*, and *VCP*, were identified for the Large White population. These genes were involved in biological processes related to mitochondrial function, protein targeting, chromatin organization, and morphogenesis.

**Conclusion:**

The findings from this study elucidate the regulatory mechanisms influencing the carcass and meat characteristics of the Neijiang and Large White pigs. The key genes could serve as potential biomarkers for enhancing breeding strategies aimed at improving pork quality.

## INTRODUCTION

Pork is a source of high-quality protein, and its quality is a major economic factor that holds an important position in the food market [1]. Meat color and pH are essential determinants of meat quality, while carcass weight (CW), backfat thickness (BFT), and eye muscle area (EMA) are also important carcass characteristics. Previous studies have identified candidate genes associated with some of these traits. For instance, Wang et al [2], through a genome-wide association study, identified eight potential candidate genes (*COL21A1, ZNF184, ZNF391, HMGA1, GRM4, NUDT3, PGM2L1*, and *PLBD2*) for carcass traits in pigs.

The rapid development of high-throughput technologies and the significant decrease in sequencing costs have led to the increased use of transcriptome analysis and differential expression analysis to identify genes linked to traits [3,4]. However, differential expression analysis focuses on individual genes and may overlook important gene-gene interactions that are relevant to target traits [5]. Weighted gene co-expression network analysis (WGCNA) addresses this by identifying genes with similar functions and regulation by constructing networks based on gene expression similarities and linking them to specific traits [5,6]. This method has been used in cancer, genetics, and brain imaging studies and has led to the identification of candidate biomarkers [1]. WGCNA is particularly useful for simultaneously identifying key genes related to multiple complex traits [5]. A previous study identified six key genes (*RAD9A, TCAP, SMYD1, PFKM, GPS2*, and *APOF*) associated with fat deposition in Songliao black and Landrace breeds using WGCNA [1]. Similarly, Wang et al [5] identified five key genes and 13 key genes influencing intramuscular fat content and meat color (CIE a*), respectively. While previous studies have applied this method to investigate gene co-expression patterns related to various traits in pigs, these studies have primarily focused on Western pigs. The Neijiang pig is a valuable genetic resource and is native to the Sichuan basin of China. The Neijiang pig is classified as a fat-type breed and is reported to have tender and flavorful meat, strong adaptability, higher disease resistance, and a slow growth rate compared to other breeds [7,8]. On the other hand, the Large White, a Western lean-type breed, is known for its high growth rate, feed conversion ratio, and lean meat percentage compared to the Neijiang pig [9]. However, the genetic mechanisms influencing carcass and meat quality traits in Chinese indigenous pig breeds like the Neijiang pig and Western breeds such as the Large White are still not well understood.

In this study, WGCNA was applied to examine gene interactions and identify key genes influencing multiple carcass and meat quality traits in Neijiang and Large White pigs. The identification of key genes will elucidate the mechanisms influencing these traits in both breeds.

## MATERIALS AND METHODS

### Animals, phenotypic characteristics, and sample collection

A total of 17 Neijiang and 22 Large White pigs with an average age of 180 days were used in this study. The Neijiang and Large White pigs had an average weight of 120 kg and 130 kg, respectively. The pigs were fed a standard commercial diet of corn and soybeans, formulated according to their body weight and adhering to Chinese national standard GB/T 5915-2020. They were transported to a commercial slaughterhouse and fasted overnight, with only *ad libitum* access to drinking water before slaughter. The animals were electrically stunned (1.3 A for 10–20 seconds) and subsequently bled. Post-slaughter and evisceration, CW and other parameters of interest were measured and recorded. The BFT and EMA (calculated as height×width×0.7) [10] were measured using a vernier caliper at the sixth and seventh ribs. Meat color lightness (L1), redness (a1), and yellowness (b1), were evaluated at 45 minutes using a Minolta CR-300 colorimeter (Minolta Camera). The muscle pH values of the longissimus dorsi muscle were recorded at 45 minutes (pH_45_) and 24 hours (pH_24_) post-mortem with a portable pH meter (model 720A; Orion Research). All assessments were conducted on the left side of the carcass. In addition, approximately 0.2 g of the longissimus dorsi muscle samples were collected and stored at −80°C for subsequent transcriptome sequencing.

### Library construction and RNA sequencing

Total RNA was extracted from 39 longissimus dorsi muscle samples, and mRNA was purified with poly-T magnetic beads. The mRNA was fragmented and converted into cDNA using random hexamer primers and reverse transcriptase. The second strand was synthesized, blunt ends created, and adaptors ligated. cDNA fragments of 370–420 bp were purified and amplified using PCR. The libraries were pooled, and DNA nanoballs were generated and sequenced using the DNBSEQ-T7 platform. The library construction was done by Novogene.

### RNA-Seq data analysis

Read quality control was performed on the raw data to obtain clean reads (Supplement 1). The clean reads were then mapped to the *Sus scrofa* reference genome (Sus scrofa11.1) using HISAT2, which was then sorted and indexed using Samtools. The genes were quantified separately for the two pig breeds using the FeatureCounts tool in the subread software, and the FPKM (fragments per kilobase per million) values were calculated [11]. Genes with FPKM values less than 1 in more than ten individuals were excluded.

### Weighted gene co-expression network analysis

The weighted gene co-expression network construction was performed using the WGCNA package (v.4.3.3) [6] in R (v.4.4.1). The datasets were filtered to include only common genes between the two breeds. The phenotype data were categorized into two groups: carcass traits (CW, BFT, and EMA) and meat traits (L1, a1, b1, pH_45_, and pH_24_). This categorization enabled a more focused examination of relationships within each trait group, leading to the identification of modules associated with these traits and breeds. The analyses were performed separately for each trait group and pig breeds using the same parameters.

Genes and samples were checked for outliers using the goodSamplesGenesMS function to ensure data quality. Using the pickSoftThreshold function [12], a soft-threshold power of 10 was chosen, and the topological overlap matrix (TOM) was computed. A reference percentile of 0.95 was employed to scale TOM values with a set seed for reproducibility. Modules were identified using dynamic tree cutting with parameters set to minModuleSize = 30, deepSplit = 2, and pamRespectsDendro = FALSE, followed by merging closely related modules based on consensus eigengene dissimilarity with a cut height of 0.25. Module-trait correlations and p-values were calculated, and the results were visualized through heatmaps. Modules were screened using an absolute correlation coefficient (|r|)≥0.3 and a p-value≤0.1 in at least one trait, and the top modules were selected based on the highest total absolute correlation. Consensus module eigengenes were recalculated to assess module relationships across the breeds.

### Protein-protein interaction network and identification of key genes

Protein-protein interaction network (PPI) was constructed to analyze the interactions between genes encoding proteins in candidate genes using the STRING database (v12.0) (https://cn.string-db.org/). Genes in the selected modules were screened based on module membership and gene significance (|MM|>0.8 and |GS|>0.3). The filtered genes were imported into the STRING database with a minimum required interaction score set to 0.40 (minimum confidence), and the resulting PPI network was visualized using Cytoscape software (v3.10.3) [13]. CytoHubba plugin (v0.1) [14] was used to rank the nodes in the PPI network using the Degree topological analysis method. Genes with high degree scores, ranking between one and two, were selected as the key genes.

### Functional enrichment analysis

Gene Ontology (GO) and Kyoto Encyclopedia of Genes and Genomes (KEGG) enrichment analyses were performed for genes in the top modules using the DAVID database (https://davidbioinformatics.nih.gov/), and p<0.05 was used as the threshold.

Enrichment analyses were also performed for the key genes using the ShinyGO database (v0.81) [15]. For GO terms, significant enrichment was determined using a false discovery rate (FDR) threshold of <0.05. However, few KEGG pathways were identified using this threshold. Therefore, the FDR threshold was adjusted to <0.1 to explore additional pathways that may be associated with the key genes.

### Feature selection and validation of key genes

To ensure the biological relevance and predictive power of the key genes, their normalized expression values were first correlated with the phenotypic traits using Pearson correlation analysis to explore associations between the genes and traits. The key genes were further analyzed using three feature selection models: Least Absolute Shrinkage and Selection Operator (LASSO) regression, Elastic Net regression, and Random Forest. LASSO regression is a method that adds a penalty to the absolute value of the coefficients, helping to simplify the model by forcing some coefficients to zero and selecting only the most relevant features. In this study, LASSO regression was performed using the glmnet R package (v4.1–8) with a Gaussian family for multi-response variables. Leave-one-out cross-validation (LOOCV) was used to identify the optimal penalty parameter (λ), with features showing nonzero coefficients considered significant [16]. Elastic Net regression blends the penalties of both LASSO and Ridge regression and is effective for datasets with highly correlated predictors. This model reduces overfitting while selecting a strong subset of relevant features. Similar to LASSO, the optimal λ was identified through LOOCV, and features with nonzero coefficients were retained as relevant contributors [16]. The Random Forest model was implemented using the randomForest package (v4.7–1.2). This ensemble method builds multiple decision trees and evaluates the importance of each feature by measuring how much its inclusion improves the model’s predictions. Importance scores were calculated using Percentage Increase in Mean Squared Error to identify the most influential genes [17]. The accuracy of these models was assessed by computing correlations between observed and predicted trait values.

### Statistical analysis

Carcass and meat quality measurements were analyzed using a two-tailed Student’s t-test in the Statistical Package for Social Science (SPSS) program (v. 22) [18] with a p-value<0.05 considered statistically significant. Data on carcass and meat quality measurements are presented as means±standard deviations. Additional analyses, including WGCNA, were conducted using the R software (v.4.4.1).

## RESULTS

### Carcass and meat quality performance

Carcass and meat quality traits are important indicators of pork quality. Significant differences were observed in both breeds (Table 1). The Large White pigs had a higher CW (99.43±10.15 kg) compared to Neijiang pigs (89.78±17.24 kg). However, the Neijiang breed had a thicker backfat (45.09±8.98 mm) than the Large White breed (22.76±3.77 mm), indicating a higher fat content as was expected. In addition, the Large White pigs had considerably larger EMA (32.01±6.26 cm^2^), reflecting greater muscle mass and leanness than Neijiang pigs (14.69±3.24 cm^2^). Furthermore, while the Large White pigs displayed a slightly higher lightness (L1) (44.70±1.51) than Neijiang pigs (43.28±2.49), Neijiang pork exhibited higher redness (a1) (10.02±2.83) and yellowness (b1) (4.47±1.20) compared to Large White pork (a1: 4.13±1.24; b1:3.26±0.49). Postmortem pH_45_ was slightly lower in Neijiang pigs (6.28± 0.10) than in Large White pigs (6.47±0.21). However, pH_24_ was higher in Neijiang pigs (5.70±0.10) than in Large White pigs (5.33±0.36).

### Weighted gene co-expression network analysis and identification of top modules

**Figure 1:**
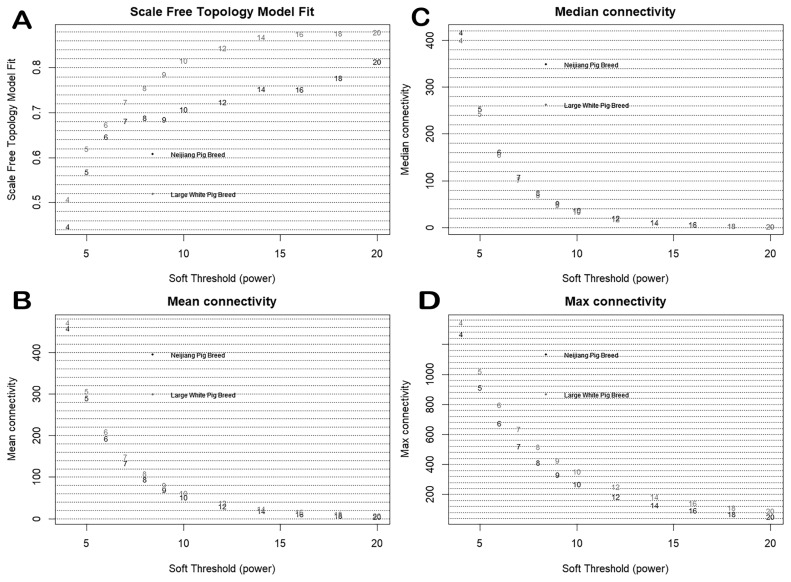
Network topology and connectivity metrics used to determine the soft-threshold power for WGCNA analysis. (A) Scale-free topology model fit. (B) Mean connectivity. (C) Median connectivity. (D) Max connectivity. The figure shows the summary of network indices (y-axes) as functions of soft-thresholding power (x-axes). Numbers in the plots indicate the corresponding powers tested. An approximate scale-free topology was achieved around a power of 10 for both datasets, based on the thresholds of 0.8 (Large White breed) and 0.7 (Neijiang breed) in panel A. Connectivity measures decrease with increasing power (panels B–D), supporting the selection of the lowest power that satisfies the scale-free criterion. Gray numbers in the plots represent the Large White breed, and black numbers represent the Neijiang breed. WGCNA, weighted gene co-expression network analysis.

**Figure 2:**
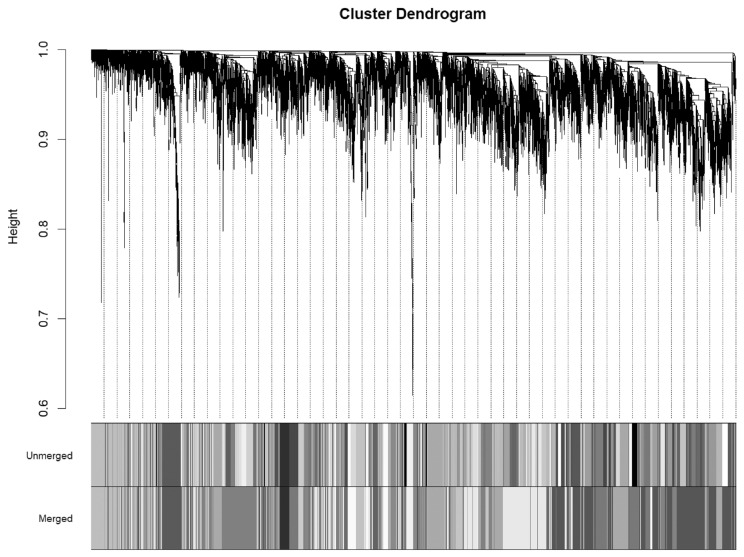
Cluster dendrogram of genes and modules. Hierarchical clustering dendrogram of genes based on topological overlap matrix (TOM) dissimilarity. The top row of color bars (Unmerged) represents the initial modules identified using dynamic tree cutting. The bottom row (Merged) shows the final module assignments after merging modules whose eigengenes were highly correlated (cut height = 0.25). The y-axis represents clustering height from hierarchical clustering. Genes with similar expression patterns cluster together into branches (modules), and color annotations below indicate module membership before and after merging.

Genes with low expression levels (FPKM<1) in more than ten individuals were excluded from the analysis, leaving 9,302 genes and 10,597 genes for the Neijiang and Large White pigs, respectively. This was further filtered to include only the genes shared between both breeds, resulting in a total of 9,249 genes, with all genes and samples passing quality checks. The scale-free topological model and mean connectivity were determined (Figures 1A, 1B), and a soft-threshold of 10 was selected as the optimal value. After dynamic tree trimming, 22 co-expression modules were identified (Figure 2), with the number of genes in the modules ranging from 51 to 1,902 (Supplement 2).

**Figure 3:**
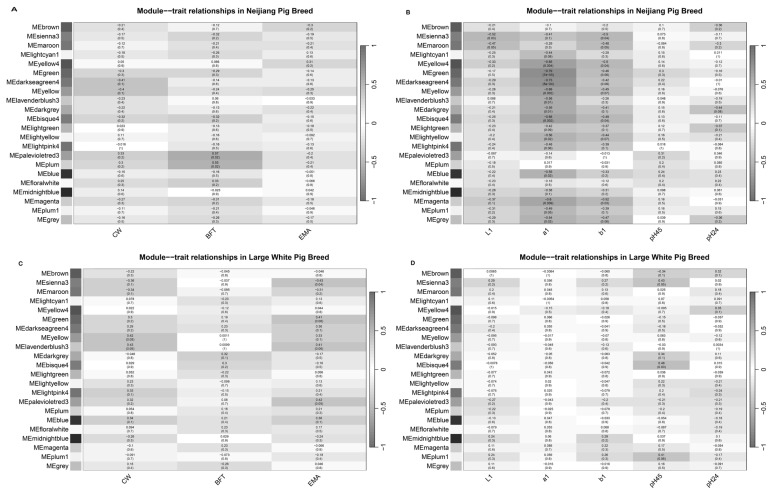
Heatmap of the correlation between modules and traits. (A) Module-trait correlation for carcass traits for the Neijiang breed. (B) Module-trait correlation for meat traits for the Neijiang breed. (C) Module-trait correlation for carcass traits for the Large White breed. (D) Module-trait correlation for meat traits for the Large White breed. Columns represent traits, and rows represent eigengene modules. The correlation coefficient values between the module eigengene and the traits, along with the p-value in parentheses, are presented in each cell. CW, carcass weight; BFT, backfat thickness; EMA, eye muscle area; L1, lightness; a1, redness; b1, yellowness; pH_45_, postmortem pH at 45 minutes; pH_24_, postmortem pH at 24 hours.

The palevioletred3 and green modules were identified as the top modules for the Neijiang dataset. The palevioletred3 module, consisting of 74 genes, showed a positive correlation with CW (r = 0.33) and BFT (r = 0.57) while exhibiting a negative correlation with EMA (r = −0.20) (Figure 3A). For meat traits, the green module, which included 1,165 genes, was negatively correlated with L1 (r = −0.17), a1 (r = −0.79), b1 (r = −0.46), and pH_24_ (r = −0.16), while positively correlated with pH_45_ (r = 0.30) (Figure 3B).

**Figure 4:**
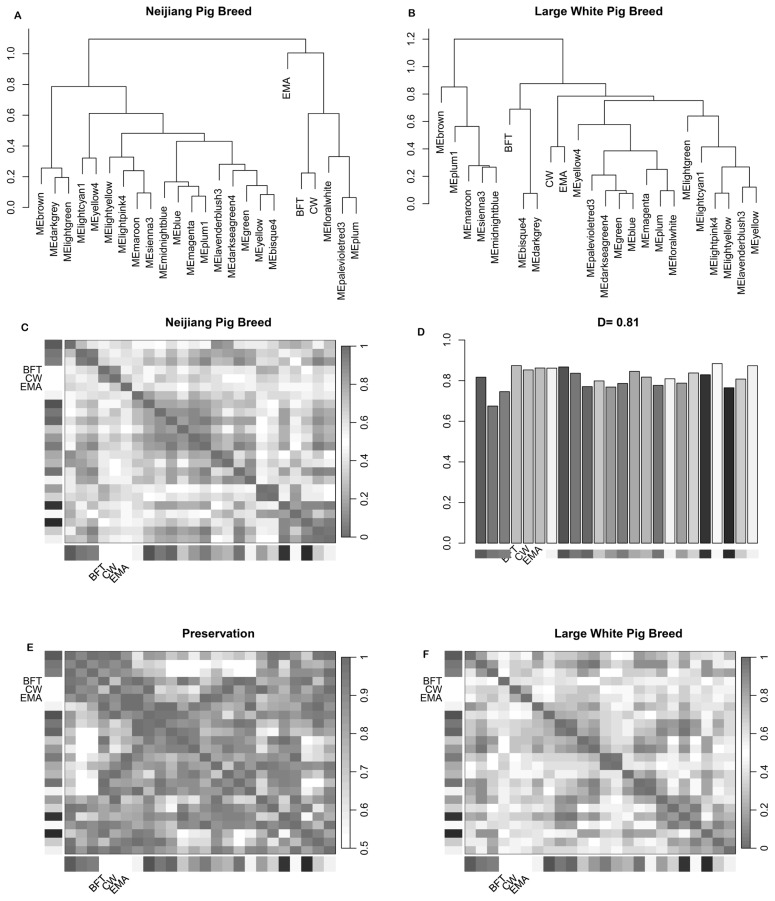
Consensus eigengene networks and their differential analysis. (A, B) Dendrogram and eigengene representation of consensus eigengene networks for Neijiang and Large White breeds, respectively. (C, F) Heatmap of eigengene adjacencies in Neijiang and Large White networks. Each row and column correspond to an eigengene tagged by consensus module colors. (D) Bar plot of preservation degree of each consensus eigengene. The height of the bar (y-axis) and each bar corresponds to the eigengene of the associated consensus module. The high-density value *D* (preserve Neijiang and Large White) = 0.81 indicates the high overall preservation between the two networks. (E) Adjacency heatmap of the preservation network between Neijiang and Large White consensus eigengene networks. The saturation of the black or gray color indicates the correlation preservation of Neijiang and Large White module eigengenes.

For the Large White population, the blue module, comprising 1,902 genes, was positively correlated with CW (r = 0.34), BFT (r = 0.21), and EMA (r = 0.36) (Figure 3C). The plum1 module, with 88 genes, was positively correlated with L1 (r = 0.24), a1 (r = 0.06), b1 (r = 0.26), and pH_45_ (r = 0.41), but negatively correlated with pH_24_ (r = −0.17) (Figure 3D). A comparative analysis was conducted on the consensus eigengene networks derived from the Neijiang and Large White datasets. These networks capture the relationships between consensus modules, quantified by eigengene correlations. The overall preservation of the two eigengene networks was high, with a preservation score of 0.81 (Figure 4). However, the consensus module-trait relationships across both breeds did not meet the predefined filtering criteria and were not analyzed further (Supplement 3).

### Functional enrichment analysis of genes in top modules

**Figure 5:**
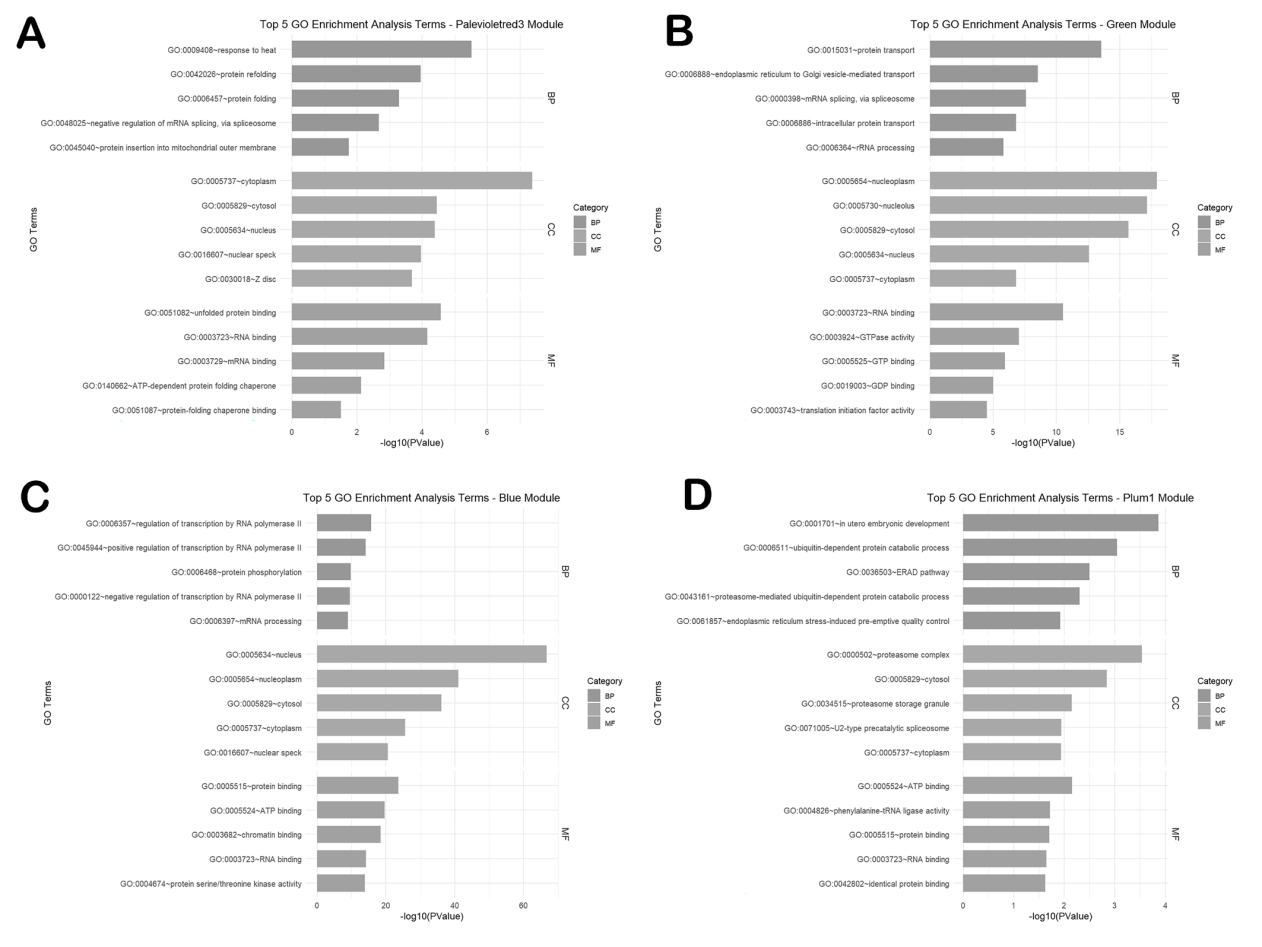
Functional enrichment analysis of genes in the selected modules. (A) Palevioletred3 module. (B) Green module. (C) Blue module. (D) Plum1 module. The top 5 significant biological processes (BP), cellular component (CC), and molecular function (MF) are presented in the plot.

Functional enrichment analysis was performed for genes in the top modules (Figure 5). In the Neijiang population, the palevioletred3 module genes were enriched in GO terms such as response to heat, protein refolding, and protein folding. Genes in the green module were enriched in protein transport, endoplasmic reticulum to Golgi vesicle-mediated transport, mRNA splicing via spliceosome, and intracellular protein transport.

In the Large White population, blue module genes were significantly enriched in biological processes, including regulation of transcription by RNA polymerase II, positive regulation of transcription by RNA polymerase II, protein phosphorylation, and negative regulation of transcription by RNA polymerase II. Furthermore, plum1 module genes were significantly enriched in processes, such as in utero embryonic development, ubiquitin-dependent protein catabolic process, ERAD pathway, and proteasome-mediated ubiquitin-dependent protein catabolic process.

**Figure 6:**
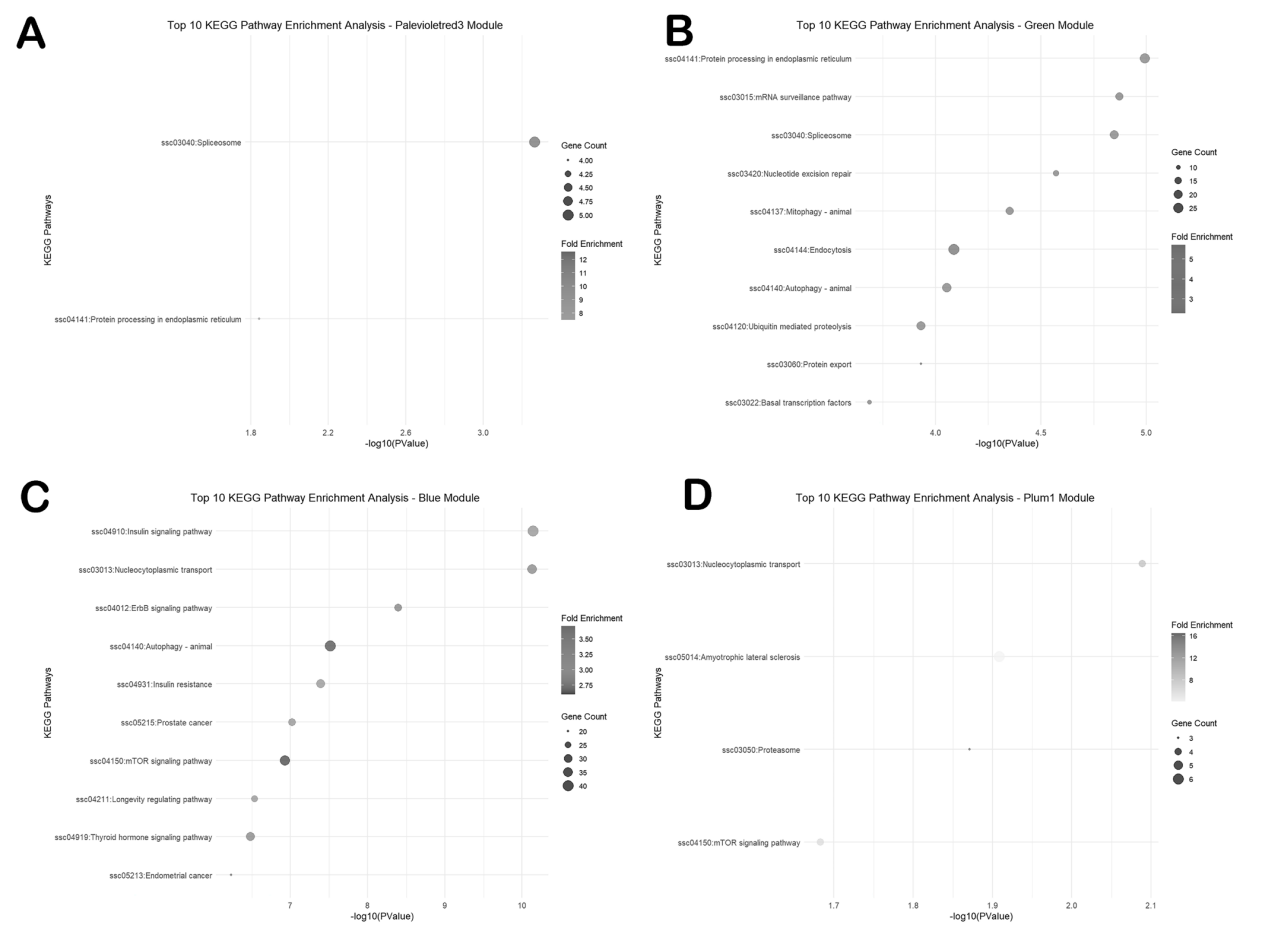
Top 10 KEGG pathway enrichment analysis of genes in the selected modules. (A) Palevioletred3 module. (B) Green module. (C) Blue module. (D) Plum1 module. KEGG, Kyoto Encyclopedia of Genes and Genomes.

The KEGG enrichment analysis was performed to explore the pathways associated with the traits (Figure 6). In the Neijiang population, the palevioletred3 module demonstrated significant enrichment in the spliceosome pathway and the protein processing in the endoplasmic reticulum pathway. The green module showed enrichment in the protein processing in the endoplasmic reticulum pathway, mRNA surveillance pathway, and spliceosome pathway. In the Large White population, the blue module exhibited significant enrichment in the insulin signaling pathway, nucleocytoplasmic transport pathway, ErbB signaling pathway, and autophagy pathway. The plum1 module demonstrated significant enrichment in the nucleocytoplasmic transport pathway, amyotrophic lateral sclerosis pathway, proteasome pathway, and mTOR signaling pathway.

### Protein-protein interaction network and key genes identification

**Figure 7:**
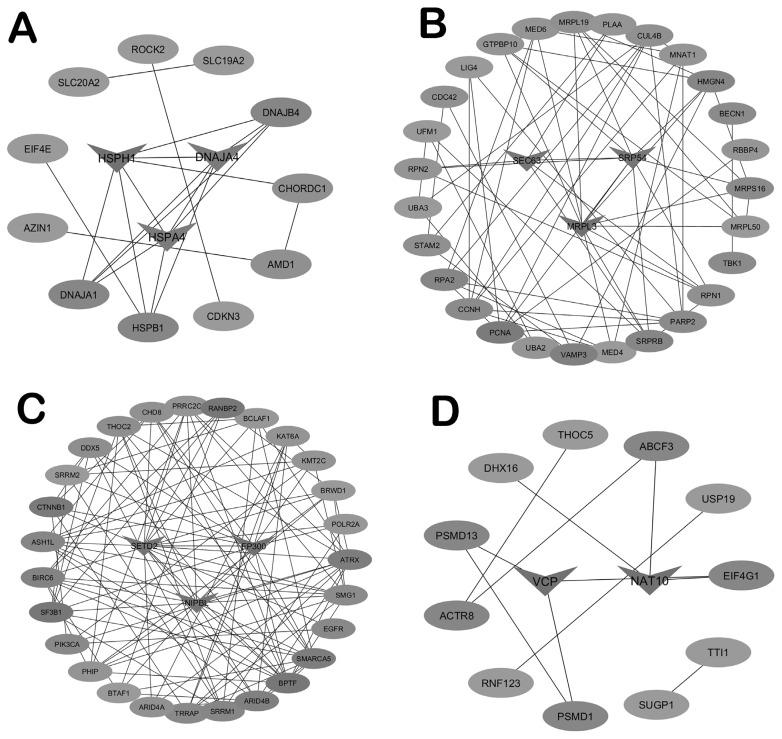
PPI network interaction of modules and key genes. (A) Palevioletred3 module. (B) Green module. (C) Blue module. (D) Plum1 module. PPI, protein-protein interaction network.

A PPI network analysis was conducted using the STRING database to explore protein interactions within selected modules. Genes were initially screened using the criteria |MM|> 0.80 and |GS|>0.30, resulting in the palevioletred3, green, blue, and plum1 modules having 27, 286, 430, and 31 genes, respectively. Genes encoding proteins with high interaction with other proteins in the modules were selected as key genes. In total, six key genes, including *HSPH1, HSPA4, DNAJA4, MRPL3, SEC63*, and *SRP54*, were identified for the Neijiang breed. Furthermore, five key genes consisting of *EP300, SETD2, NIPBL, NAT10*, and *VCP*, were also identified in the Large White dataset. These key genes are important for understanding the biological processes influencing the traits of interest. Figure 7 presents the PPI network and key genes of the top modules.

The key genes in the Neijiang dataset were significantly enriched in biological processes, such as outer mitochondrial membrane organization, protein insertion into mitochondrial outer membrane, SRP-dependent cotranslational protein targeting to membrane translocation, and post-translational protein targeting to membrane translocation. Key genes in the Large White breed were enriched in the regulation of protein localization to chromatin, protein localization to chromatin, and face, head, and body morphogenesis.

**Figure 8:**
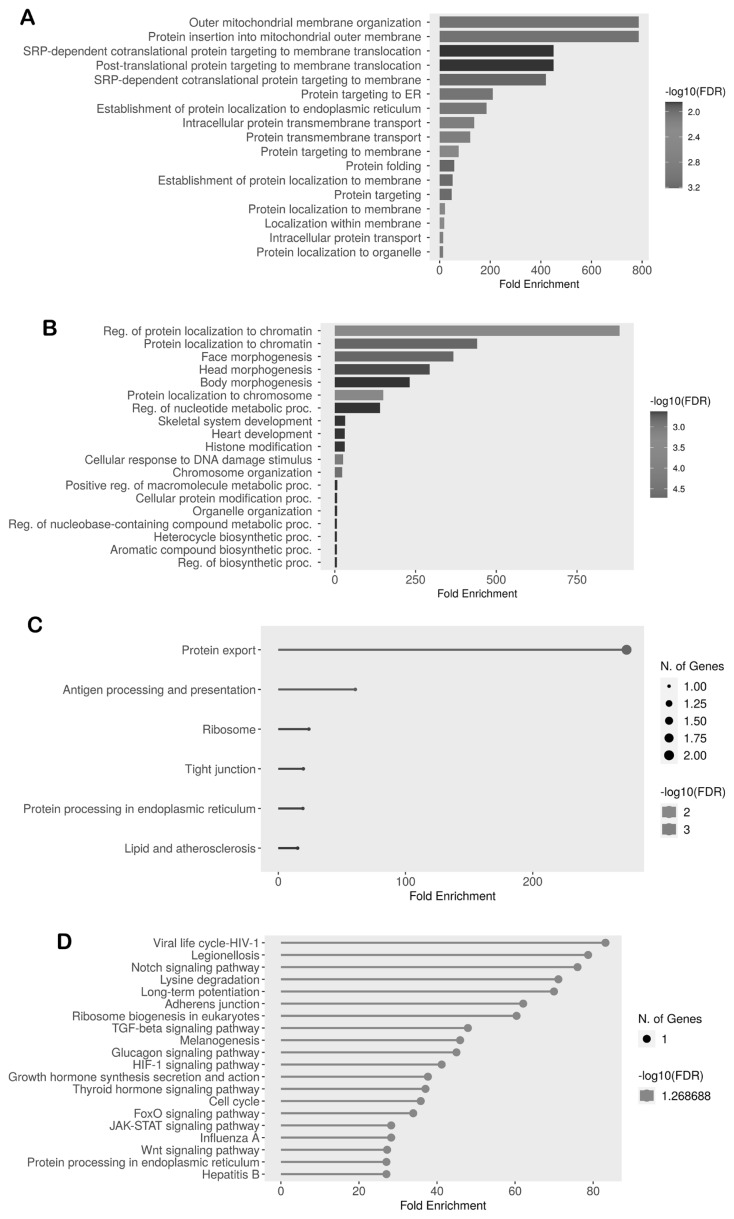
GO and KEGG pathway enrichment analysis of key genes associated with carcass and meat traits in the Neijiang and Large White pig breeds. (A) GO biological process enrichment analysis of key genes in the Neijiang breed. (B) GO biological process enrichment analysis of key genes in the Large White breed. (C) KEGG pathway enrichment analysis of key genes in the Neijiang breed. (D) KEGG pathway enrichment analysis of key genes in the Large White breed. GO, Gene Ontology; KEGG, Kyoto Encyclopedia of Genes and Genomes.

KEGG pathway enrichment analysis revealed protein export, antigen processing and presentation, ribosome, and tight junction pathways for key genes in the Neijiang breed. Key genes for the Large White breed were enriched in pathways such as viral life cycle-HIV-1, Legionellosis, notch signaling pathway, and lysine degradation (Figure 8).

**Figure 9:**
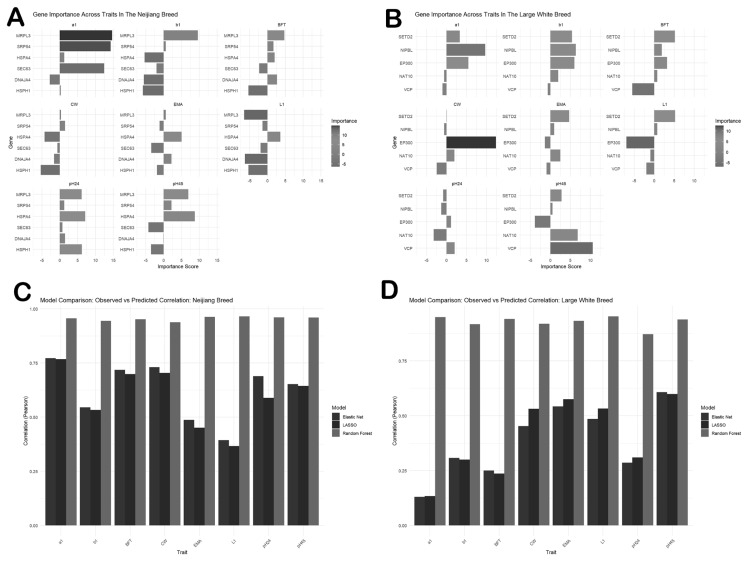
Importance scores of key genes and model comparison of LASSO, Elastic Net, and Random Forest models. (A) Importance scores of key genes for the Neijiang breed. (B) Importance scores of key genes for the Large White breed. (C) Model comparison of LASSO, Elastic Net, and Random Forest models for the Neijiang dataset. (D) Model comparison of LASSO, Elastic Net, and Random Forest models for the Large White dataset.

To validate the key genes identified through WGCNA and PPI network analysis, we employed three complementary machine learning approaches: LASSO regression, Elastic Net regression, and Random Forest. These methods were applied to analyze the relationship between the key genes and traits. The key genes were first correlated with these traits, and the analysis revealed that the genes were significantly correlated with most of the traits (Supplement 4). LASSO regression, which is known for its strength in feature selection and reducing overfitting, identified five key genes (*SEC63, HSPA4, DNAJA4, SRP54*, and *MRPL3*) in the Neijiang dataset. In the Large White dataset, four key genes (*NAT10, EP300, SETD2*, and *NIPBL*) were identified. Elastic Net regression identified additional genes consisting of *HSPH1* and *VCP* for the Neijiang and Large White datasets, respectively (Supplements 5–8). Random Forest analysis assigned importance scores for the genes to support their relevance. Genes with the highest importance score were deemed the most influential in predicting traits. Among the identified genes, *SRP54, MRPL3*, and *HSPA4* were the top predictors of the carcass and meat traits in the Neijiang pig breed, while *EP300*, *SETD2*, *NIPBL*, and *VCP* were among the top predictors of traits in the Large White breed (Figures 9A, 9B; Supplements 9–12). The top key genes and their importance scores are presented in Table 2.

The predictive performance of the models was evaluated by calculating the correlation coefficients between predicted trait values and observed values. The LASSO model demonstrated an accuracy ranging from 0.37 to 0.77 and 0.13 to 0.60 for the Neijiang and Large White datasets, respectively. Elastic Net showed moderate predictive accuracy, with correlation coefficients ranging from 0.39 to 0.77 and 0.13 to 0.61 for the Neijiang and Large White breeds, respectively. The Random Forest model demonstrated the highest accuracy, with correlation coefficients ranging from 0.94 to 0.96 and 0.87 and 0.95 for the Neijiang and Large White breeds, respectively, indicating a strong predictive power (Figures 9C, 9D, Supplement 13).

## DISCUSSION

Meat quality and carcass characteristics are important factors and are largely influenced by consumer demand for high-quality and affordable products. This demand has led to the integration of these traits into breeding programs to improve pork quality [19]. This study observed differences in the carcass and meat quality characteristics between Neijiang and Large White pigs. The Large White pigs had a higher CW and EMA, which indicates a high meat yield and leanness. Also, Neijiang pigs had thicker backfat, indicating higher fat content, which can influence meat flavor and juiciness. Higher redness and yellowness, which are often associated with perceived freshness and quality, were observed in the Neijiang pork. The Neijiang pigs also had higher pH values, which can positively impact meat tenderness. Similar phenotypic distinctions between indigenous and commercial pig breeds, such as Ghungroo and Large White pigs, have been previously reported, with indigenous breeds generally exhibiting superior meat quality traits but lower lean yield [20]. However, comprehending the biological and molecular regulatory mechanisms influencing these unique traits is essential. Therefore, this study utilized WGCNA to identify key genes and molecular mechanisms regulating meat quality and carcass traits in the Neijiang and Large White pig breeds.

This study identified 22 consensus modules through WGCNA analysis. In the Neijiang population, the palevioletred3 module demonstrated significant enrichment in biological processes related to stress responses, including response to heat. Other processes included protein refolding and protein folding. These processes are involved in maintaining normal cellular function and preserving protein integrity, especially under stressful conditions. Heat shock proteins (*HSPs*) assist cells to recognize and refold damaged proteins or target them for degradation, effectively removing proteins that can no longer function properly. Under stress, *HSP* levels rise, promoting the synthesis and maturation of new proteins to replace those affected by stress. This increase not only aids in protein repair but also helps maintain cell viability by inhibiting apoptosis [21]. The presence of genes involved in these processes in Neijiang pigs also suggests possible adaptive mechanisms that may help maintain or enhance meat quality traits, particularly under stressful conditions. Additionally, the green module’s enrichment in protein transport and mRNA splicing via spliceosome may be important in regulating the molecular mechanisms behind muscle development and quality. The efficient transport of proteins and the precise regulation of gene expression are necessary for the development of desirable carcass traits, including backfat and muscle area [22].

In the Large White population, genes in the blue module were enriched in transcriptional regulation by RNA polymerase II. Previous studies on meat quality have identified transcription regulation processes, including negative and positive regulation by RNA polymerase II [23,24]. Positive regulation enhances gene expression through the activation of transcription factors that bind to promoters or enhancers, while negative regulation acts to suppress gene activity. This balance is necessary for maintaining cellular homeostasis and preventing overactive or inappropriate gene expression [25]. These regulatory mechanisms likely function in controlling gene expression within muscle tissues and influence meat quality and carcass traits. Additionally, the plum1 module was involved in in-utero embryonic development and ubiquitin-dependent protein catabolic process, pointing to their potential in early growth and protein turnover [26].

The key genes further elucidate the molecular mechanisms influencing carcass and meat traits in the two breeds. The six key genes from the Neijiang dataset were predominantly involved in mitochondrial function and protein targeting, indicating their role in maintaining cellular homeostasis and energy metabolism. Mitochondria play a role in oxygen consumption, energy metabolism, and apoptotic processes. These processes influence myoglobin levels, oxidative stress, meat tenderness, fat oxidation, and protein oxidation, which affect traits such as meat color, tenderness, and flavor [27]. The presence of heat shock proteins (*HSPs*) like *HSPH1* and *HSPA4* suggests a protective stress response in muscle tissues. This response helps preserve muscle cell integrity under various physiological conditions, which are essential for desirable meat traits [28].

The five key genes identified in the Large White breed were enriched in chromatin organization and morphogenesis processes. The regulation of protein localization to chromatin reveals the importance of transcriptional regulation in the development of the traits. These genes are involved in functions such as DNA repair, gene expression regulation, and cellular signaling pathways, which are important for the proper development of physical traits such as body and head morphology and may influence the carcass and meat quality characteristics in the Large White pigs [27–29].

LASSO, Elastic Net regression, and Random Forest analyses were performed to validate and prioritize the key genes. LASSO selected five genes for the Neijiang and four for Large White, while Elastic Net identified six and five genes, respectively, for the two breeds, which further strengthens the relevance of the identified key genes. The Random Forest model was then used to assess the importance of the key genes in predicting traits. In the Nejiang pig breed, *SRP54* was the top predictor for CW. *SRP54* is involved in targeting secretory proteins to the endoplasmic reticulum, which is linked to protein synthesis and secretion [30]. Similarly, *MRPL3*, a mitochondrial ribosomal protein essential for mitochondrial protein synthesis, was identified as a key gene associated with multiple traits, including BFT and meat color (a1 and b1). These findings are consistent with a previous study suggesting that higher mitochondrial activity is associated with increased BFT and higher pH [31]. Mitochondrial activity has been reported to influence beef color through oxygen consumption and metmyoglobin reduction [32,33]. *HSPA4* was also associated with multiple traits (EMA, L1, pH_45_, and pH_24_). *HSPA4’s* function in stress response, protein folding, and maintenance of muscle function through regulation of skeletal muscle autophagy and apoptosis may regulate meat quality characteristics [34]. Higher expression of heat shock proteins has been reported to be associated with better meat water-holding capacity and resistance to stress-induced quality deterioration in indigenous pigs than in exotic pigs [20].

In the Large White breed, the *EP300* gene was associated with CW. *EP300* functions as a histone and lysyl acetyltransferase that influences adipocyte differentiation, lipid storage, and lipid metabolism. Research has shown that *EP300* regulates lipid metabolism and energy homeostasis in pigs [35]. High expression of *EP300* has also been linked to increases in body weight, body mass index, total cholesterol levels, low-density lipoprotein cholesterol levels, and triglyceride levels [36]. *SETD2*, the top predictor for BFT, EMA, and L1, is involved in DNA repair and gene regulation and contributes to metabolic reprogramming during myogenesis. Its role in muscle energy metabolism may influence meat quality [37,38]. The *NIPBL* gene was the main predictor of a1 and b1. *NIPBL* facilitates interactions between enhancers and promoters and is involved in embryo development and embryonic morphogenesis. It has also been linked with adipogenesis, limb development, BFT, and other traits in livestock, suggesting that it can contribute to carcass composition and meat quality [39]. In addition, *VCP* was identified as the primary predictor for pH_45_ and pH_24_. As a highly conserved AAA-ATPase, *VCP* is integral to ubiquitin-dependent protein quality control pathways. Its multifaceted roles in protein degradation and various cellular processes indicate that *VCP* may be important for regulating muscle development [40].

The identified key genes reveal the potential mechanisms influencing the meat and carcass traits in the Neijiang and Large White pigs. Integrating them into genomic selection, marker-assisted breeding programs, and nutritional interventions that influence the activity of these genes could further improve carcass and meat quality characteristics in pigs.

## CONCLUSION

This study revealed differences in carcass and meat quality traits between the Neijiang and Large White pigs. Through WGCNA analysis, we identified six key genes in the Neijiang breed and five key genes in the Large White breed, which are involved in processes such as mitochondrial function, protein targeting, chromatin organization, and morphogenesis. The identified key genes elucidate the molecular mechanisms underlying the carcass and meat characteristics of both breeds and could serve as potential biomarkers.

## Figures and Tables

**Table 1:** Carcass and meat quality characteristics of the Neijiang and Large White pig breeds

Parameters	Neijiang breed	Large White breed	p-value
CW (kg)	89.78±17.24	99.43±10.15	0.051
BFT (mm)	45.09±8.98	22.76±3.77	0.001
EMA (cm^2^)	14.69±3.24	32.01±6.26	0.001
L1	43.28±2.49	44.70±1.51	0.034
a1	10.02±2.83	4.13±1.24	0.001
b1	4.47±1.20	3.26±0.49	0.001
pH_45_	6.28±0.10	6.47±0.21	0.001
pH_24_	5.70±0.10	5.33±0.36	0.001

Data are presented as the mean±standard deviation.

CW, carcass weight; BFT, backfat thickness; EMA, eye muscle area; L1, lightness; a1, redness; b1, yellowness; pH_45_, postmortem pH at 45 minutes; pH_24_, postmortem pH at 24 hours.

**Table 2:** Top key genes and their importance scores based on Random Forest analysis

Breed	Gene	Trait	Importance score
Neijiang	*SRP54*	CW	1.49
Neijiang	*MRPL3*	BFT	4.76
Neijiang	*MRPL3*	a1	14.68
Neijiang	*MRPL3*	b1	9.64
Neijiang	*HSPA4*	EMA	5.06
Neijiang	*HSPA4*	L1	3.66
Neijiang	*HSPA4*	pH_45_	8.73
Neijiang	*HSPA4*	pH_24_	7.12
Large White	*EP300*	CW	12.37
Large White	*SETD2*	BFT	5.22
Large White	*SETD2*	EMA	4.69
Large White	*SETD2*	L1	5.29
Large White	*NIPBL*	a1	9.72
Large White	*SETD2*	b1	6.40
Large White	*VCP*	pH45	10.63
Large White	*VCP*	pH24	2.02

CW, carcass weight; BFT, backfat thickness; EMA, eye muscle area; L1, lightness; a1, redness; b1, yellowness; pH_45_, postmortem pH at 45 minutes; pH_24_, postmortem pH at 24 hours.

## SUPPLEMENTARY MATERIAL

**Supplement 1.** Summary of RNA-seq data.

**Supplement 2.** Modules and gene numbers.

**Supplement 3.** Consensus relationship of consensus module eigengenes and traits across the Neijiang and Large White dataset.

**Supplement 4.** Correlation analyses between key genes and traits.

**Supplement 5.** Genes selected by LASSO regression analysis for the Neijiang dataset.

**Supplement 6**. Genes selected by LASSO regression analysis for the Large White dataset.

**Supplement 7.** Genes selected by Elastic Net analysis for the Neijiang dataset.

**Supplement 8.** Genes selected by Elastic Net analysis for the Large White dataset.

**Supplement 9.** Genes selected by Random Forest analysis for the Neijiang dataset.

**Supplement 10.** Genes selected by Random Forest analysis for the Large White dataset.

**Supplement 11.** Importance score for Random Forest model - Neijiang dataset.

**Supplement 12.** Importance score for Random Forest model - Large White dataset.

**Supplement 13.** Model comparison: Observed vs Predicted correlation.

## References

[1] Xing K, Liu H, Zhang F (2021). Identification of key genes affecting porcine fat deposition based on co-expression network analysis of weighted genes. J Anim Sci Biotechnol.

[2] Wang H, Wang X, Yan D (2022). Genome-wide association study identifying genetic variants associated with carcass backfat thickness, lean percentage and fat percentage in a four-way crossbred pig population using SLAF-seq technology. BMC Genomics.

[3] Sodhi SS, Park WC, Ghosh M (2014). Comparative transcriptomic analysis to identify differentially expressed genes in fat tissue of adult Berkshire and Jeju native pig using RNA-seq. Mol Biol Rep.

[4] Xing K, Zhu F, Zhai L (2016). Identification of genes for controlling swine adipose deposition by integrating transcriptome, whole-genome resequencing, and quantitative trait loci data. Sci Rep.

[5] Wang B, Hou L, Yang W (2024). Construction of a co-expression network affecting intramuscular fat content and meat color redness based on transcriptome analysis. Front Genet.

[6] Langfelder P, Horvath S (2008). WGCNA: an R package for weighted correlation network analysis. BMC Bioinf.

[7] Li Y (2021). Comparing of backfat microRNAomes of Landrace and Neijiang pig by high-throughput sequencing. Genes Genomics.

[8] Tao X, Kong FJ, Liang Y (2023). Screening of candidate genes related to differences in growth and development between Chinese indigenous and Western pig breeds. Physiol Genomics.

[9] Guo X, Qin B, Yang X (2019). Comparison of carcass traits, meat quality and expressions of MyHCs in muscles between Mashen and large white pigs. Ital J Anim Sci.

[10] Wang D, Chen G, Chai M (2022). Effects of dietary protein levels on production performance, meat quality and flavor of fattening pigs. Front Nutr.

[11] Liao Y, Smyth GK, Shi W (2013). featureCounts: an efficient general purpose program for assigning sequence reads to genomic features. Bioinformatics.

[12] Zhang B, Horvath S (2005). A general framework for weighted gene co-expression network analysis. Stat Appl Genet Mol Biol.

[13] Shannon P, Markiel A, Ozier O (2003). Cytoscape: a software environment for integrated models of biomolecular interaction networks. Genome Res.

[14] Chin CH, Chen SH, Wu HH, Ho CW, Ko MT, Lin CY (2014). cytoHubba: identifying hub objects and sub-networks from complex interactome. BMC Syst Biol.

[15] Ge SX, Jung D, Yao R (2019). ShinyGO: a graphical gene-set enrichment tool for animals and plants. Bioinformatics.

[16] Ogutu JO, Schulz-Streeck T, Piepho HP (2012). Genomic selection using regularized linear regression models: ridge regression, lasso, elastic net and their extensions. BMC Proc.

[17] Alsahaf A, Azzopardi G, Ducro B, Hanenberg E, Veerkamp RF, Petkov N (2018). Prediction of slaughter age in pigs and assessment of the predictive value of phenotypic and genetic information using random forest. J Anim Sci.

[18] IBM (2013). IBM SPSS statistics for windows. Ver. 22.0.

[19] Green HE, Oliveira HR, Alvarenga AB (2024). Genomic background of biotypes related to growth, carcass and meat quality traits in Duroc pigs based on principal component analysis. J Anim Breed Genet.

[20] Parkunan T, Das AK, Banerjee D (2017). Changes in expression of monocarboxylate transporters, heat shock proteins and meat quality of large white Yorkshire and Ghungroo pigs during hot summer period. Asian-Australas J Anim Sci.

[21] Silva DBS, Fonseca LFS, Pinheiro DG (2020). Spliced genes in muscle from Nelore cattle and their association with carcass and meat quality. Sci Rep.

[22] Arikawa LM, Mota LFM, Schmidt PI (2024). Genome-wide scans identify biological and metabolic pathways regulating carcass and meat quality traits in beef cattle. Meat Sci.

[23] Meng Q, Wang K, Liu X (2017). Identification of growth trait related genes in a Yorkshire purebred pig population by genome-wide association studies. Asian-Australas J Anim Sci.

[24] Zhao L, Zhou L, Hao X (2021). Identification and characterization of circular RNAs in association with the deposition of intramuscular fat in Aohan fine-wool sheep. Front Genet.

[25] Smith CL, Eppig JT (2009). The mammalian phenotype ontology: enabling robust annotation and comparative analysis. Wiley Interdiscip Rev Syst Biol Med.

[26] Baldarelli RM, Smith CL, Ringwald M, Richardson JE, Bult CJ (2024). Mouse Genome Informatics: an integrated knowledgebase system for the laboratory mouse. Genetics.

[27] Zou B, Jia F, Ji L, Li X, Dai R (2024). Effects of mitochondria on postmortem meat quality: characteristic, isolation, energy metabolism, apoptosis and oxygen consumption. Crit Rev Food Sci Nutr.

[28] Ijaz M, Li X, Hou C, Hussain Z, Zhang D (2024). Role of heat-shock proteins in the determination of postmortem metabolism and meat quality development of DFD meat. Foods.

[29] Villaseñor R, Baubec T (2021). Regulatory mechanisms governing chromatin organization and function. Curr Opin Cell Biol.

[30] Gowda K, Black SD, Moeller I, Sakakibara Y, Liu MC, Zwieb C (1998). Protein SRP54 of human signal recognition particle: cloning, expression, and comparative analysis of functional sites. Gene.

[31] Molinero E, Pena RN, Estany J, Ros-Freixedes R (2025). Association between mitochondrial DNA copy number and production traits in pigs. J Anim Breed Genet.

[32] Cheong A, Lingutla R, Mager J (2020). Expression analysis of mammalian mitochondrial ribosomal protein genes. Gene Expr Patterns.

[33] Ramanathan R, Mancini RA (2018). Role of Mitochondria in beef color: a review. Meat Muscle Biol.

[34] Elkenani M, Barakat AZ, Held T (2022). Heat shock protein A4 ablation leads to skeletal muscle myopathy associated with dysregulated autophagy and induced apoptosis. J Transl Med.

[35] Yu H, Yang Z, Wang J (2024). Identification of key genes and metabolites involved in meat quality performance in Qinchuan cattle by WGCNA. J Integr Agric.

[36] Martínez-Pinteño A, Gassó P, Prohens L (2021). Identification of EP300 as a key gene involved in antipsychotic-induced metabolic dysregulation based on integrative bioinformatics analysis of multi-tissue gene expression data. Front Pharmacol.

[37] Carvalho S, Vítor AC, Sridhara SC (2014). SETD2 is required for DNA double-strand break repair and activation of the p53-mediated checkpoint. eLife.

[38] Wiedner HJ, Torres EV, Blue RE (2022). SET domain containing 2 (SETD2) influences metabolism and alternative splicing during myogenesis. FEBS J.

[39] Yin S, Song G, Gao N (2023). Identifying genetic architecture of carcass and meat quality traits in a Ningxiang indigenous pig population. Genes.

[40] Ahlstedt BA, Ganji R, Raman M (2022). The functional importance of VCP to maintaining cellular protein homeostasis. Biochem Soc Trans.

