# Supplementary material for: Comprehensive co-expression analysis reveals candidate regulatory genes associated with carcass and meat quality traits in Neijiang and Large White pigs
Source: Anim Biosci. 2025 Jun 24;38(12):2568–83. doi: 10.5713/ab.25.0259 (PMC12580783; doi:10.5713/ab.25.0259)
Supplement: Supplementary file 1 [file ab-25-0259-Supplementary-1.pdf]

Supplement 1. Summary of RNA-seq data

Sample	Obtained Reads	Obtained Base(G)	Q20 (%)	Q30 (%)	GC (%)
NJP1	133772008	20065801200	99.3	97.73	54.82
NJP2	130374814	19556222100	99.09	97.03	53.88
NJP3	123222378	18483356700	99.08	97.01	55.62
NJP4	122322352	18348352800	99.23	97.51	54.32
NJP5	67607740	10141161000	98.88	96.33	53.53
NJP6	112208990	16831348500	99.08	97.02	53.6
NJP7	121877788	18281668200	99.23	97.52	54.25
NJP8	141393138	21208970700	99.27	97.63	55.33
NJP9	127737900	19160685000	99.24	97.56	55.15
NJP10	106562458	15984368700	99.08	97	54.16
NJP11	147363190	22104478500	99.3	97.74	53.78
NJP12	139299204	20894880600	99.29	97.71	54.81
NJP13	141934752	21290212800	99.29	97.72	55.18
NJP14	150008442	22501266300	99.33	97.84	54.8
NJP15	79686948	11953042200	99.25	97.6	55.51
NJP16	113125656	16968848400	99.09	97.04	53.68
NJP17	140569954	21085493100	99.34	97.86	54.66
LW1	54997874	8249681100	98.58	96.28	53.05
LW2	149292796	22393919400	99.16	97.29	53.69
LW3	120286962	18043044300	99.2	97.39	54.75
LW4	111933712	16790056800	99.11	97.11	53.71
LW5	104146420	15621963000	99.24	97.52	51.98
LW6	134913374	20237006100	99.16	97.28	52.26
LW7	125203956	18780593400	99.19	97.37	53.5
LW8	136324124	20448618600	99.17	97.31	53.33
LW9	131314810	19697221500	98.95	96.59	52.52
LW10	139668968	20950345200	99.1	97.08	54.25
LW11	129592744	19438911600	99.03	96.87	53.05
LW12	122905296	18435794400	99.31	97.75	54.2
LW13	114453218	17167982700	99.09	97.04	54.28
LW14	113513520	17027028000	99.25	97.55	54.55
LW15	117815020	17672253000	98.94	96.56	54.36
LW16	110804280	16620642000	98.98	96.67	52.93
LW17	106181452	15927217800	99.03	96.82	53.32
LW18	117684900	17652735000	99.09	97.02	54.5
LW19	101404676	15210701400	99.23	97.51	55.54
LW20	118770888	17815633200	99.12	97.14	54.24
LW21	129931488	19489723200	99.14	97.18	53.42
LW22	127362308	19104346200	99.24	97.53	53.17
