# Supplementary material for: Comprehensive co-expression analysis reveals candidate regulatory genes associated with carcass and meat quality traits in Neijiang and Large White pigs
Source: Anim Biosci. 2025 Jun 24;38(12):2568–83. doi: 10.5713/ab.25.0259 (PMC12580783; doi:10.5713/ab.25.0259)
Supplement: Supplementary file 2 [file ab-25-0259-Supplementary-2.pdf]

Supplement 2. Modules and gene numbers

moduleColors	Freq
bisque4	802
blue	1902
brown	314
darkgrey	133
darkseagreen4	178
floralwhite	161
green	1165
grey	934
lavenderblush3	174
lightcyan1	84
lightgreen	148
lightpink4	276
lightyellow	256
magenta	780
maroon	69
midnightblue	158
palevioletred3	74
plum	51
plum1	88
sienna3	103
yellow	1344
yellow4	55
