# Supplementary material for: Comprehensive co-expression analysis reveals candidate regulatory genes associated with carcass and meat quality traits in Neijiang and Large White pigs
Source: Anim Biosci. 2025 Jun 24;38(12):2568–83. doi: 10.5713/ab.25.0259 (PMC12580783; doi:10.5713/ab.25.0259)
Supplement: Supplementary file 3 [file ab-25-0259-Supplementary-3.pdf]

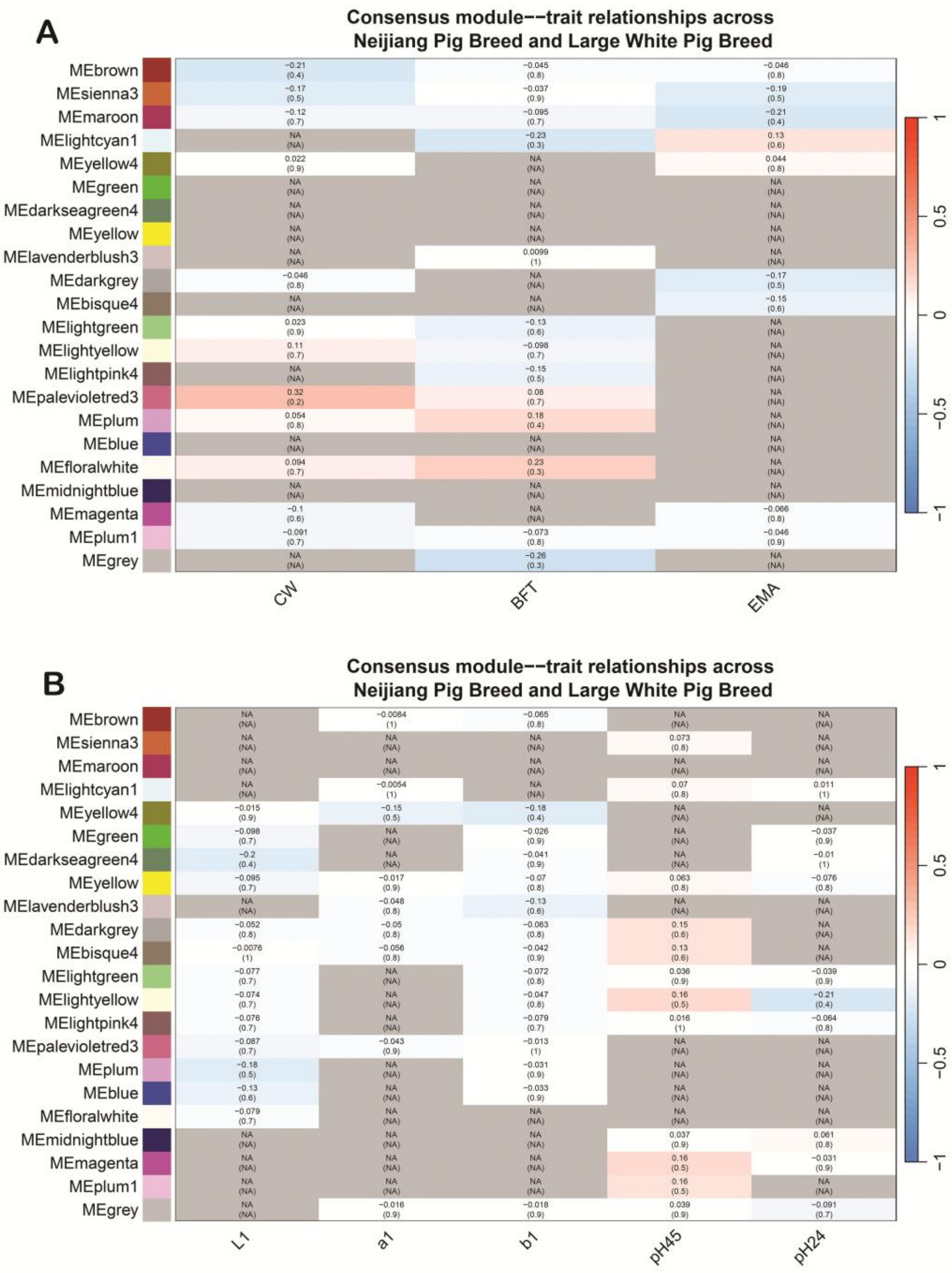

Supplement 3. Consensus relationship of consensus module eigengenes and traits across the Neijiang and Large White dataset. (A) Consensus module-trait correlation for carcass trait category. (B) Consensus module-trait correlation for meat trait category. **Note.** Columns represent traits, and rows represent eigengene modules. Red denotes positive correlation, while blue denotes negative correlation. The correlation coefficient values between the consensus module eigengene and the traits, along with the p-value in parentheses, are presented in each cell. Missing (NA) entries indicate that the correlations in the Neijiang and Large White datasets have opposite signs, and no consensus can be formed.
