# Supplementary material for: Comprehensive co-expression analysis reveals candidate regulatory genes associated with carcass and meat quality traits in Neijiang and Large White pigs
Source: Anim Biosci. 2025 Jun 24;38(12):2568–83. doi: 10.5713/ab.25.0259 (PMC12580783; doi:10.5713/ab.25.0259)
Supplement: Supplementary file 4 [file ab-25-0259-Supplementary-4.pdf]

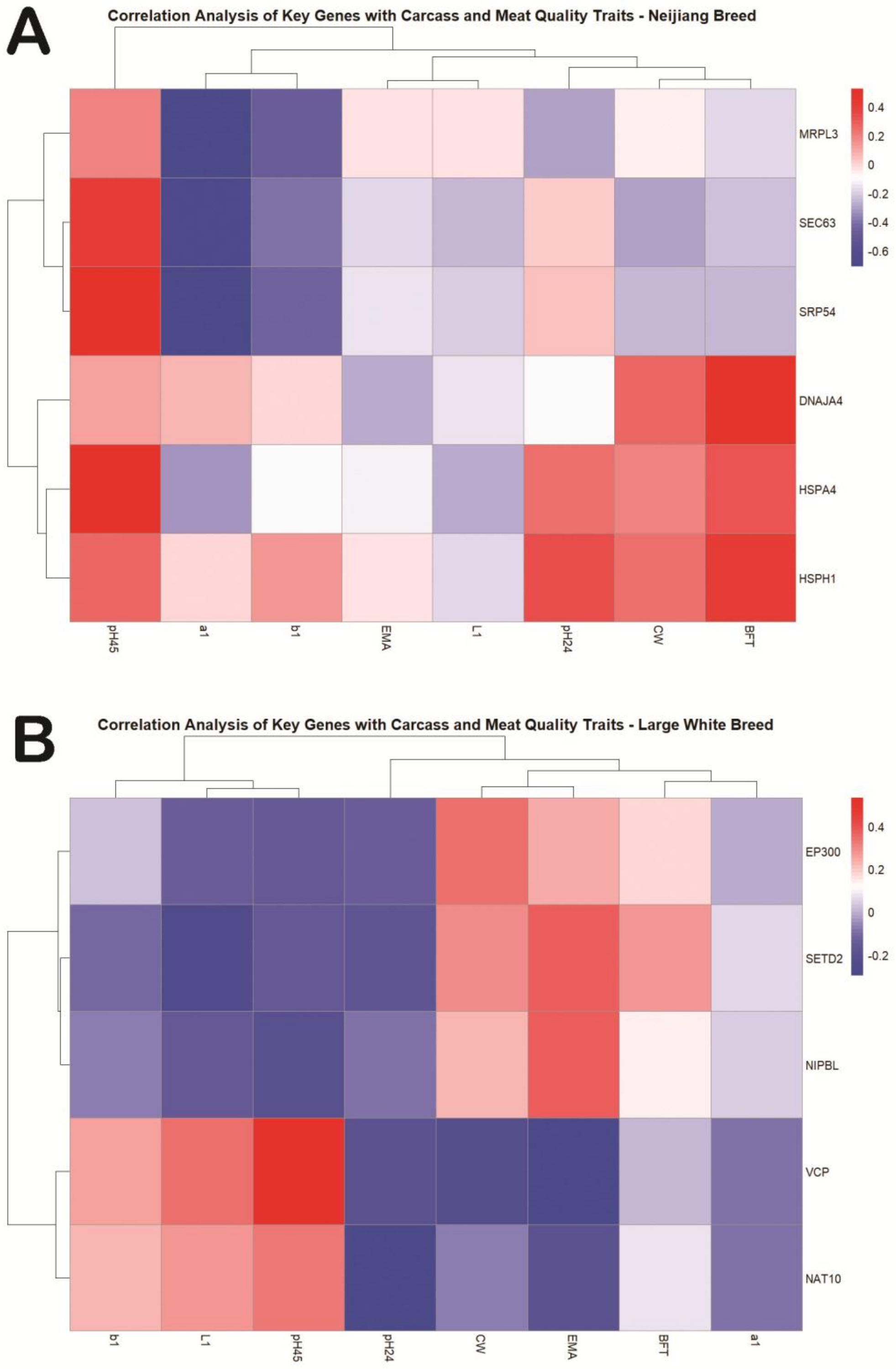

Supplement 4. Correlation analyses between key genes and traits. (A) Neijiang dataset. (B) Large White dataset.
