# Supplementary material for: Comprehensive co-expression analysis reveals candidate regulatory genes associated with carcass and meat quality traits in Neijiang and Large White pigs
Source: Anim Biosci. 2025 Jun 24;38(12):2568–83. doi: 10.5713/ab.25.0259 (PMC12580783; doi:10.5713/ab.25.0259)
Supplement: Supplementary file 7 [file ab-25-0259-Supplementary-7.pdf]

Supplement 7. Genes selected by Elastic Net analysis for the Neijiang dataset

	CW	BFT	EMA	L1	a1	b1	pH_{45}	pH_{24}
1	SEC63	SEC63	SEC63	SEC63	SEC63	SEC63	SEC63	SEC63
2	HSPA4	HSPA4	HSPA4	HSPA4	HSPA4	HSPA4	HSPA4	HSPA4
3	DNAJA4	DNAJA4	DNAJA4	DNAJA4	DNAJA4	DNAJA4	DNAJA4	DNAJA4
4	SRP54	SRP54	SRP54	SRP54	SRP54	SRP54	SRP54	SRP54
5	HSPH1	HSPH1	HSPH1	HSPH1	HSPH1	HSPH1	HSPH1	HSPH1
6	MRPL3	MRPL3	MRPL3	MRPL3	MRPL3	MRPL3	MRPL3	MRPL3
