# Supplementary material for: Comprehensive co-expression analysis reveals candidate regulatory genes associated with carcass and meat quality traits in Neijiang and Large White pigs
Source: Anim Biosci. 2025 Jun 24;38(12):2568–83. doi: 10.5713/ab.25.0259 (PMC12580783; doi:10.5713/ab.25.0259)
Supplement: Supplementary file 9 [file ab-25-0259-Supplementary-9.pdf]

Supplement 9. Genes selected by Random Forest analysis for the Neijiang dataset

	CW	BFT	EMA	L1	a1	b1	pH_{45}	pH_{24}
1	SRP54	MRPL3	HSPA4	HSPA4	MRPL3	MRPL3	HSPA4	HSPA4
2	MRPL3	DNAJA4	DNAJA4	SRP54	SRP54	SRP54	MRPL3	HSPH1
3	SEC63	HSPA4	MRPL3	SEC63	SEC63	SEC63	SRP54	MRPL3
4	DNAJA4	SRP54	SRP54	HSPH1	HSPA4	HSPA4	DNAJA4	DNAJA4
5	HSPA4	SEC63	HSPH1	DNAJA4	HSPH1	DNAJA4	HSPH1	SRP54
6	HSPH1	HSPH1	SEC63	MRPL3	DNAJA4	HSPH1	SEC63	SEC63
