# Supplementary material for: Comprehensive co-expression analysis reveals candidate regulatory genes associated with carcass and meat quality traits in Neijiang and Large White pigs
Source: Anim Biosci. 2025 Jun 24;38(12):2568–83. doi: 10.5713/ab.25.0259 (PMC12580783; doi:10.5713/ab.25.0259)
Supplement: Supplementary file 10 [file ab-25-0259-Supplementary-10.pdf]

Supplement 10. Genes selected by Random Forest analysis for the Large White dataset

	CW	BFT	EMA	L1	a1	b1	pH_{45}	pH_{24}
1	EP300	SETD2	SETD2	SETD2	NIPBL	NIPBL	VCP	VCP
2	NAT10	EP300	NAT10	NIPBL	EP300	EP300	NAT10	EP300
3	SETD2	NIPBL	NIPBL	NAT10	SETD2	SETD2	SETD2	SETD2
4	NIPBL	NAT10	VCP	VCP	NAT10	NAT10	NIPBL	NIPBL
5	VCP	VCP	EP300	EP300	VCP	VCP	EP300	NAT10
