# Supplementary material for: Comprehensive co-expression analysis reveals candidate regulatory genes associated with carcass and meat quality traits in Neijiang and Large White pigs
Source: Anim Biosci. 2025 Jun 24;38(12):2568–83. doi: 10.5713/ab.25.0259 (PMC12580783; doi:10.5713/ab.25.0259)
Supplement: Supplementary file 11 [file ab-25-0259-Supplementary-11.pdf]

Supplement 11. Importance score for Random Forest model - Neijiang dataset

Gene	Importance	Trait
SEC63	-0.6619	CW
HSPA4	-4.2315	CW
DNAJA4	-1.5652	CW
SRP54	1.4868	CW
HSPH1	-5.3347	CW
MRPL3	0.3657	CW
SEC63	-2.3009	BFT
HSPA4	2.0318	BFT
DNAJA4	2.6904	BFT
SRP54	1.6647	BFT
HSPH1	-5.2844	BFT
MRPL3	4.7636	BFT
SEC63	-3.4307	EMA
HSPA4	5.0606	EMA
DNAJA4	2.2404	EMA
SRP54	-1.1179	EMA
HSPH1	-1.8335	EMA
MRPL3	0.6295	EMA
SEC63	-1.8877	L1
HSPA4	3.6553	L1
DNAJA4	-6.3107	L1
SRP54	-1.3612	L1
HSPH1	-5.3122	L1
MRPL3	-6.4725	L1
SEC63	12.4559	a1
HSPA4	1.2573	a1
DNAJA4	-2.7914	a1
SRP54	14.2718	a1
HSPH1	0.3058	a1
MRPL3	14.6817	a1
SEC63	-1.9564	b1
HSPA4	-5.3316	b1
DNAJA4	-5.4800	b1
SRP54	0.6407	b1
HSPH1	-5.7631	b1
MRPL3	9.6392	b1
SEC63	-4.2498	pH45
HSPA4	8.7296	pH45
DNAJA4	-0.0496	pH45
SRP54	2.2377	pH45
HSPH1	-3.5151	pH45
MRPL3	6.9394	pH45
SEC63	0.7283	pH24
HSPA4	7.1176	pH24
DNAJA4	1.5078	pH24

SRP54	1.3089	pH24
HSPH1	6.1834	pH24
MRPL3	6.1749	pH24
