# Supplementary material for: Comprehensive co-expression analysis reveals candidate regulatory genes associated with carcass and meat quality traits in Neijiang and Large White pigs
Source: Anim Biosci. 2025 Jun 24;38(12):2568–83. doi: 10.5713/ab.25.0259 (PMC12580783; doi:10.5713/ab.25.0259)
Supplement: Supplementary file 12 [file ab-25-0259-Supplementary-12.pdf]

Supplement 12. Importance score for Random Forest model - Large White dataset

Gene	Importance	Trait
VCP	-2.4250	CW
NAT10	1.9971	CW
EP300	12.3711	CW
SETD2	0.0926	CW
NIPBL	-0.6045	CW
VCP	-5.4339	BFT
NAT10	0.7848	BFT
EP300	3.2050	BFT
SETD2	5.2223	BFT
NIPBL	1.9410	BFT
VCP	-0.9520	EMA
NAT10	2.5165	EMA
EP300	-1.3771	EMA
SETD2	4.6860	EMA
NIPBL	0.9733	EMA
VCP	-1.9376	L1
NAT10	-0.9537	L1
EP300	-6.9608	L1
SETD2	5.2849	L1
NIPBL	0.7862	L1
VCP	-1.0094	a1
NAT10	-0.5899	a1
EP300	5.4594	a1
SETD2	3.3604	a1
NIPBL	9.7224	a1
VCP	-0.6522	b1
NAT10	1.9493	b1
EP300	6.0237	b1
SETD2	5.4392	b1
NIPBL	6.4005	b1
VCP	10.6296	pH45
NAT10	6.8840	pH45
EP300	-3.8329	pH45
SETD2	2.8059	pH45
NIPBL	0.5446	pH45
VCP	2.0180	pH24
NAT10	-3.1893	pH24
EP300	1.1189	pH24
SETD2	-0.8054	pH24
NIPBL	-1.3110	pH24
