# Supplementary material for: Comprehensive co-expression analysis reveals candidate regulatory genes associated with carcass and meat quality traits in Neijiang and Large White pigs
Source: Anim Biosci. 2025 Jun 24;38(12):2568–83. doi: 10.5713/ab.25.0259 (PMC12580783; doi:10.5713/ab.25.0259)
Supplement: Supplementary file 13 [file ab-25-0259-Supplementary-13.pdf]

Supplement 13. Model comparison: Observed vs Predicted correlation

	LASSO - Neijiang	LASSO - Large White	Elastic Net - Neijiang	Elastic Net - Large White	Random Forest - Neijiang	Random Forest - Large White
CW	0.7038	0.5309	0.7302	0.4526	0.9375	0.9186
BFT	0.6983	0.2361	0.7184	0.2503	0.9511	0.9406
EMA	0.4501	0.5751	0.4863	0.5423	0.9626	0.9318
L1	0.3663	0.5324	0.3933	0.4851	0.9641	0.9519
a1	0.7668	0.1337	0.7711	0.1304	0.9559	0.9490
b1	0.5325	0.2996	0.5446	0.3079	0.9433	0.9164
pH45	0.6439	0.5982	0.6527	0.6075	0.9591	0.9385
pH24	0.5886	0.3090	0.6882	0.2852	0.9606	0.8711
